# The Impairments of α-Synuclein and Mechanistic Target of Rapamycin in Rotenone-Induced SH-SY5Y Cells and Mice Model of Parkinson’s Disease

**DOI:** 10.3389/fnins.2019.01028

**Published:** 2019-09-24

**Authors:** Mahesh Ramalingam, Yu-Jin Huh, Yun-Il Lee

**Affiliations:** Well Aging Research Center, DGIST, Daegu, South Korea

**Keywords:** rotenone, SH-SY5Y, α-synuclein, mitofusin, stereotaxic, mTOR

## Abstract

Parkinson’s disease (PD) is characterized by selective degeneration of dopaminergic (DAergic) neurons in the substantia nigra pars compacta (SNpc). α-synuclein (α-syn) is known to regulate mitochondrial function and both PINK1 and Parkin have been shown to eliminate damaged mitochondria in PD. Mechanistic target of rapamycin (mTOR) is expressed in several distinct subcellular compartments and mediates the effects of nutrients, growth factors, and stress on cell growth. However, the contributions of these various regulators to DAergic cell death have been demonstrated mainly in culture with serum, which is known to dramatically influence endogenous growth rate and toxin susceptibility through nutrient and growth factor signaling. Therefore, we compared neurotoxicity induced by the mitochondrial inhibitor rotenone (ROT, 5 or 10 μM for 24 h) in SH-SY5Y cells cultured with 10% fetal bovine serum (FBS), 1% FBS, or 1% bovine serum albumin (BSA, serum-free). In addition, C57BL/6J mice were injected with 12 μg ROT into the right striatum, and brains examined by histology and Western blotting 2 weeks later for evidence of DAergic cell death and the underlying signaling mechanisms. ROT dose-dependently reduced SH-SY5Y cell viability in all serum groups without a significant effect of serum concentration. ROT injection also significantly reduced immunoreactivity for the DAergic cell marker tyrosine hydroxylase (TH) in both the mouse striatum and SNpc. Western blotting revealed that ROT inhibited TH and Parkin expression while increasing α-syn and PINK1 expression in both SH-SY5Y cells and injected mice, consistent with disruption of mitochondrial function. Moreover, expression levels of the mTOR signaling pathway components mTORC, AMP-activated protein kinase (AMPK), ULK1, and ATG13 were altered in ROT-induced PD. Further, serum level influenced mTOR signaling in the absence of ROT and the changes in response to ROT. Signs of endoplasmic reticulum (ER) stress and altered expression of tethering proteins mediating mitochondria-associated ER contacts (MAMs) were also altered concomitant with ROT-induced neurodegeneration. Taken together, this study demonstrates that complex mechanism involving mitochondrial dysfunction, altered mTOR nutrient-sensing pathways, ER stress, and disrupted MAM protein dynamics are involved in DAergic neurodegeneration in response to ROT.

## Introduction

Parkinson’s disease (PD) is an age-related neurodegenerative disorder (NDD) characterized by progressive loss of dopaminergic (DAergic) neurons in the substantia nigra (SN) pars compacta (SNpc) along with intracellular aggregation of α-synuclein (α-syn) in structures known as Lewy bodies (LBs) (Lee and Trojanowski, 2006). Neurons use multiple feedback controls to regulate metabolism in response to nutrients and other signals. Neurons depend on oxidative phosphorylation (OXPHOS) for most energy needs, a process that consumes oxygen and glucose to generate energy-storing ATP (Hsu and Sabatini, 2008). This process relies on electron flow via electron transport chain (ETC) components in the inner mitochondrial membrane, culminating in the reduction of oxygen in the matrix, and the generation of a membrane potential across the inner membrane that is exploited to convert ADP to ATP (Whitworth and Pallanck, 2017).

Rotenone (ROT) is a naturally occurring insecticide, pesticide, and piscicide extracted from the roots of plants of the genera *Lonchocarpus* and *Derris*. It is highly lipophilic and therefore easily crosses all biological membranes including blood-brain barrier (Martinez and Greenamyre, 2012). The mitochondrial toxin ROT is widely used to induced a PD-like pathology in culture cells and experimental animals. ROT impairs OXPHOS by inhibiting mitochondrial ETC complex I (reduced nicotinamide adenine dinucleotide-ubiquinone reductase), leading to reduced ATP production and the formation of reactive oxygen species (ROS) that can induce oxidative stress (Duty and Jenner, 2011; Martinez and Greenamyre, 2012). The major advantages of ROT treatment for PD modeling are its ability to induce α-syn-positive cytoplasmic inclusions in nigral neurons resembling LBs (Betarbet et al., 2000) and progressive neurodegeneration accompanied by PD-like motor and non-motor symptoms (Johnson and Bobrovskaya, 2015).

In addition to external stressors, nutrient signals, growth factors, and cell energy balance (a product of mitochondrial function) are major regulators of cellular growth, proliferation, and survival in health and disease (Yang et al., 2019). However, the signaling pathways underlying nutrient effects in PD are largely unknown. Mechanistic/mammalian target of rapamycin (mTOR) is a serine-threonine kinase that controls several important aspects of mammalian cellular function through nutrient signal transduction (Saxton and Sabatini, 2017). It exists in two distinct multiprotein complexes, mTORC1 and mTORC2 (Switon et al., 2017), each with its own unique subunit composition and functions. The mTORC1 complex comprised of mTOR, mLST-8, FKBP38, Deptor, PRAS40, and rapamycin-sensitive adaptor protein of mTOR (Raptor) regulates cell growth, proliferation, and metabolism (Bai and Jiang, 2010), whereas mTORC2 comprised of mTOR, mSIN1, mLST8, and the rapamycin-insensitive subunit Rictor (Laplante and Sabatini, 2009) controls cell survival and cell-cycle dependent cytoskeleton assembly. Each complex also utilizes distinct cofactors and substrates for regulation of these processes according to nutrient and energy status. Collectively, these two complexes regulate multiple physiologic processes (Morrison Joly et al., 2016), such as axonal growth, neuronal development and survival, and synaptic plasticity, thereby contributing to learning and memory (Jaworski and Sheng, 2006; Swiech et al., 2008). The AMP-activated protein kinase (AMPK) is another widely recognized energy-sensing serine/threonine kinase (Mihaylova and Shaw, 2011). AMPK responds to oxidative stress and critically involved in NDDs (Jiang et al., 2013). In addition, AMPK-driven mTOR downregulation serves as a turn-off switch of the cellular anabolic program (Swiech et al., 2008). AMPK directly regulates the autophagy-associated kinase ULK1 through phosphorylation under nutrient signaling. Moreover, mTORC1 also phosphorylates ULK1 at Ser757 and affect the interaction between ULK1 and AMPK (Kim et al., 2011; Shang et al., 2011). Therefore, these mutually interacting protein signaling pathways may sense and integrate countless stimuli from nutrients and growth factors to direct normal cellular processes and pathogenic processes in NDDs like PD (Linke et al., 2017).

Endoplasmic reticulum (ER) stress also contributes to multiple pathophysiological processes in NDDs (Lavoie et al., 2011). Autophagy, Ca^2+^ homeostasis, lipid metabolism, mitochondrial ATP production, mitochondrial transport and biogenesis, ER stress, and the unfolded protein response (UPR) are fundamental cellular processes dependent on direct communication between the ER and mitochondria (Paillusson et al., 2017). Approximately 5–20% of the mitochondrial surface is closely apposed (within ∼10–30 nm) to ER membranes, forming specialized regions termed mitochondria-associated ER membranes (MAMs) (Paillusson et al., 2017). The MAM contains chaperones, oxidoreductases, calcium channels, calcium buffering proteins, and regulators of lipid metabolism. Thus, this subcellular compartment is likely involved in metabolic regulation by orchestrating protein folding, lipid synthesis, calcium buffering (Patergnani et al., 2011), and oxidation/reduction (Vance, 2014). MAM formation is dynamically regulated by tethering proteins between these organelles, such as glucose-regulated protein 75 (GRP75), mitofusin 1 (Mfn1), and Mfn2 (Ma et al., 2017). α-syn disrupts the MAM, which affects cellular exchange between the two organelles. Thus, MAM dysfunction may be a potential molecular mechanism linking α-syn to PD (Paillusson et al., 2017).

Despite extensive research efforts, it is still largely unclear how nutrients regulate the protein signaling pathways relevant to NDDs such as PD. Fetal bovine serum (FBS) has been used for mammalian cell culture to promote growth, differentiation, and survival (Piletz et al., 2018). However, serum is not a physiological fluid *in vivo*, and has been shown to induce aberrant cell growth characteristics, alter phenotype, and suppress neurotoxicity *in vitro* (Pirkmajer and Chibalin, 2011; Tekkatte et al., 2011). As many of these investigations have employed cells cultured in serum, the pathogenic pathways underlying the ROT-induced PD phenotype may differ substantially from PD pathogenesis *in vivo*. Therefore, it is critical to examine the effects of serum-containing cell culture media on cellular models of PD. Here we examined ROT toxicity in SH-SY5Y neuroblastoma cells under three different cell culture conditions: (1) 10% FBS, (2) low (1%) FBS, and (3) serum-free medium (0% FBS) containing 1% bovine serum albumin (BSA) [used as synthetic serum as previously reported (Ramalingam and Kim, 2016)]. We also compared results to stereotaxic ROT injection in C57BL/6J mice. This study aimed to elucidate the molecular mechanism underlying ROT-induced toxicity, specifically the distinct contributions of mTOR/AMPK, and ER-mitochondrial tethering pathways.

## Materials and Methods

### Chemicals, Reagents and Antibodies

Dulbecco’s modified Eagle’s medium (DMEM), penicillin streptomycin (Pen Strep), trypsin-EDTA, and FBS were purchased from Welgene (South Korea). Rotenone (R8875), dimethyl sulfoxide (DMSO; D2650), Avertin (2,2,2-Tribromoethanol; T48402) were purchased from Sigma-Aldrich (St. Louis, MO, United States). All other chemicals and reagents were from commercial suppliers and of the highest purity available. Plastic materials were purchased from SPL Life Science (SPL, Seoul, South Korea). The primary and secondary antibodies used in this study were tabled in Supplementary Table 1.

### Cell Culture and Treatment

The human neuroblastoma cell line SH-SY5Y (CRL-2266) was obtained from ATCC (Manassas, VA, United States) and maintained in DMEM supplemented with 10% FBS, Pen Strep (100 U/ml; 100 mg/ml), and 2mM L-glutamine, at 37°C in a humidified atmosphere containing 5% CO_2_/95% air. Confluent cultures were washed with phosphate-buffered saline (PBS), detached with 0.25% trypsin-EDTA solution, reseeded as 1 × 10^5^ cells/ml of DMEM containing 10% FBS or 1% FBS or 1% BSA and used for experiments after overnight incubation. SH-SY5Y cells were incubated with the absence or presence of ROT for 24 h. Combining floating cells in the medium and adherent cells detached by trypsinization and subjected to cell counting and Western blotting.

### Cell Counting and Cell Morphology

After treated with ROT or solvent control (DMSO) at the indicated concentrations for 24 h, phase contrast images were taken using microscope Olympus CKX41 equipped with a camera. Damaged and deplated floating cells in the medium and adherent cells detached by trypsinization were combined and subjected to trypan blue cell counting method. Surviving cells, which cannot be stained with trypan blue dye, were counted under microscope. The cell count assay was performed in triplicates and expressed as a percentage (%) of control.

### Preparation of Total Cell Lysates and Immunoblotting

After treated with indicated concentrations of ROT or DMSO for 24 h, cells were harvested by scraping with media, pelleted and washed twice with PBS. Then, exposed to RIPA buffer (25 mM Tris–HCl (pH 7.6), 150 mM NaCl, 1% Nonidet P-40, 0.251% sodium deoxycholate, 1% sodium dodecyl sulfate (SDS): Thermo Fisher Scientific, United States) supplemented with protease and phosphatase inhibitors cocktail (Thermo Fisher Scientific, United States) and incubated for 30 min in ice. Lysates were centrifuged at 13,000 rpm for 20 min at 4°C and the supernatants were collected as total cell lysate. Protein concentrations were determined by BCA method (Kit). Proteins (30 μg) were separated on 6–12% SDS-polyacrylamide gels and transferred to PVDF membranes (Millipore, Bellerica, MA, United States). The membranes were washed with Tris buffered saline (TBS; 10 mM Tris–HCl, 150 mM NaCl, pH 7.5) containing 0.5% (v/v) Tween 20 (TBST) followed by blocking with 5% (v/v) non-fat dried milk solution prepared in TBST and then incubated overnight with primary antibodies at 4°C. The antibodies used are listed in Supplementary Table 1. After this, membranes were exposed to secondary antibodies conjugated to horseradish peroxidase for 2∼3 h at room temperature and further washed thrice with TBST. The immunoreactivity was detected by the luminol-based chemiluminescence (ECL) system. Equal protein loading was assessed by the expression level of β-actin. Densitometric analysis was performed using ImageJ (National Institute of Health, Bethesda, MD, United States) software.

### Triton-X-100-Soluble and -Insoluble Fractionation

Following ROT toxicity for 24 h, SH-SY5Y cells were lysed on ice in RIPA buffer containing protease and phosphatase inhibitors with 1% Triton-X-100 for 30 min. Lysates were centrifuged at 12,000 rpm for 20 min at 4°C and the supernatants were collected as Triton-X-100-souble fraction. The cell pellets were washed with PBS then dissolved in the RIPA buffer containing protease and phosphatase inhibitors with 1% Triton-X-100 and 2% SDS and sonicated for 10 s and used as Triton-X-100-insoluble fraction. Protein samples were immunoblotted as described above.

### Animals and Stereotaxic Surgery

Five-week-old C57BL/6J male mice were purchased from DBL (South Korea) were housed at room temperature under 12 h ligh/dark cycle. Food and water were provided *ad libitum* for 1 week before intrastriatal surgery. All animal experiments were approved by the Ethical Committee of Animal Research of DGIST, Daegu, South Korea accordance with international guidelines (DGIST-IACUC-18010204-01). Mice underwent unilateral stereotaxic surgery under injectable 2.5% Avertin anesthesia. A hole was drilled in the skull and a cannula inserted at following stereotaxic coordinates at AP + 1.0, ML −2.5 from bregma and DV −3.0 below dura in the right striatum, and 12 μg of freshly prepared ROT (dissolved in 2 μl DMSO) was infused (0.2 μl/min for 10 min for infusion with 5 min for diffusion). Control animals were injected with vehicle DMSO. Fourteen days after surgery, mice were anesthetized with avertin and perfused.

### Immunohistochemistry

Mice were sacrificed by terminal anesthesia and transcardially perfused with 50 ml PBS followed by 50 ml 4% paraformaldehyde (PFA). Brains were rapidly removed, post-fixed in 4% PFA for 24 h and stored in a 30% sucrose solution for 48 h or more. Serial coronal sections (35 μm) were cut using a freezing sledge microtome and a 1:4 series of sections was used for all quantitative immunohistochemistry. For immunohistochemical analyses, blocking of non-specific secondary antibody binding (using 3% normal horse serum in PBS with 0.2% Triton X-100 at room temperature for 1 h), sections were incubated overnight at room temperature with the primary antibody of TH (1:1000) diluted in PBS with 0.2% Triton X-100. Sections were then incubated in a biotinylated secondary antibody for 1 h [horse anti-mouse (1:2000, Vector, United Kingdom)] followed by 1 h incubation in streptavidin-biotin-horseradish peroxidase solution (Vector, United Kingdom). Sections were developed in 0.5% solution of diaminobenzidine (DAB) tetrahydrochloride (Sigma, Ireland) and mounted on microscope slides coverslipped using DPX mountant (BDH chemicals, United Kingdom). Immunostained sections were photographed and the total number of TH positive neurons in the SNpc was determined using the Optical fractionator probe in Stereo Investigator software (MicroBrightfield, Williston, VT, United States). All stereological counting was performed in a blinded manner to mice treatments.

### Cell Lysates Preparation and Immunoblotting

After fourteen days of surgery, mice were anesthetized with avertin and perfused with PBS. Mice brain subregions of midbrain (MB) and striatum (ST) were located following procedures described previously (Jackson-Lewis and Przedborski, 2007). The lysates were prepared and Western blotting done as mentioned above.

### Statistical Analysis

Data are expressed as mean ± standard error mean (SEM). The significance level of treatment effects was determined using one-way analysis of variance (ANOVA) followed by Tukey’s multiple comparison test (*in vitro*; three or more groups) or an paired/unpaired two-tailed Student’s *t*-test (*in vivo*; two-group comparisons). A probability of <5% (*p* < 0.05) was considered to be statistically significant. GraphPad Prism 5.0 software (La Jolla, CA) was used for data analyses and preparation of all graphs.

## Results

### Rotenone-Induced Death in SH-SY5Y Cells

**FIGURE 1:**
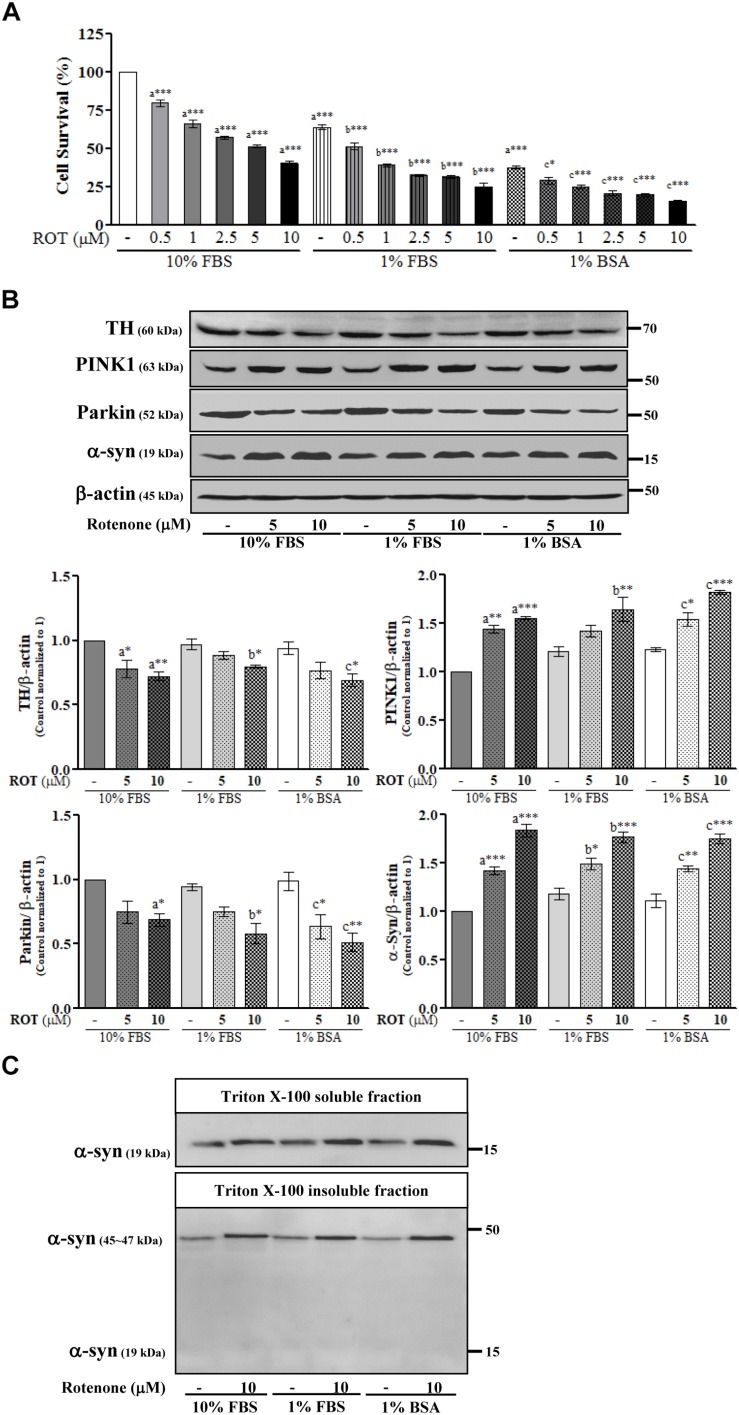
SH-SY5Y cells were seeded as 1 × 10^5^ cells/ml of DMEM containing 10% FBS or 1% FBS or 1% BSA and used for experiments after overnight incubation. Cells were incubated with the absence or presence of different concentrations of ROT (0, 0.5, 1, 2.5, 5, and 10 μM) for 24 h and assessed for trypan blue assay **(A)** or TH, PINK1, Parkin, α-syn and β-actin by Western blotting **(B)**. In addition, cells were fractionated into 1% Triton X-100 soluble and insoluble fractions and analyzed for α-syn by Western blotting **(C)**. Each picture is a representative of three independent experiments. Data are mean ± SEM of three independent experiments and analyzed by one-way of variance (ANOVA) followed by Tukey’s *post hoc* test. Statistical significance: _a_compared with 10% FBS control; _b_compared with 1% FBS control; _c_compared with 1% BSA control; ^∗^*p* < 0.05, ^∗∗^*p* < 0.01, and ^∗∗∗^*p* < 0.001.

SH-SY5Y cells were cultured in the same medium containing different serum concentrations and then treated with ROT (0, 0.5, 1, 2.5, 5, and 10 μM) for 24 h. Microscopic examination revealed that the majority of cells were damaged and deplated following 5 and 10 μM ROT treatment in 1% FBS and 1% BSA culture medium (Supplementary Figure 1). Trypan blue cell viability assays combining both floating and adherent cells detached by trypsinization revealed little proliferation in 1% FBS or 1% BSA (*p* < 0.001) compared to control 10% FBS culture media. ROT dose-dependently increased cell death in all groups after 24 h (all *p* < 0.001; Figure 1A). However, cell death was substantially greater in the low-serum and no-serum groups.

### Rotenone Alters TH, Parkin, PINK1, and α-Syn Expression in SH-SY5Y Cells

To further evaluate ROT toxicity in SH-SY5Y cells, we measured expression of tyrosine hydroxylase (TH), the rate-limiting enzyme for dopamine (DA) synthesis. Indeed, ROT significantly reduced TH protein expression in all serum groups (*p* < 0.05; Figure 1B). Moreover, ROT treatment (10 μM for 24 h) significantly increased expression of PINK1 (*p* < 0.001 in 10% FBS, *p* < 0.01 in 1% FBS, *p* < 0.001 in 1% BSA) and decreased expression of Parkin (*p* < 0.05 in 10% and 1% FBS, *p* < 0.01 in 1% BSA) as evidenced by Western blotting (Figure 1B), suggesting effects on mitochondrial function, quality control, and mitophagy.

Parkinson’s disease is characterized by the presence of abnormal intracellular α-syn inclusions. ROT treatment increased α-syn in total cell lysates from all three serum concentrations groups (*p* < 0.001; Figure 1B), suggesting that ROT reduces SH-SY5Y DAergic neuron viability by promoting α-syn accumulation. Separate Western blot analyzes of the Triton-X100-soluble and insoluble lysate fractions, which are thought to include bioavailable and aggregated α-syn proteins, respectively revealed an increase of the oligomeric form in the Triton-X100-insoluble fraction and an increase of the monomeric form in the Triton-X100-soluble fraction. Thus, ROT may induce SH-SY5Y cell death through enhanced α-syn production and ensuing aggregation (Figure 1C).

### Rotenone Alters mTORC and AMPK Expression Levels in SH-SY5Y Cells

**FIGURE 2:**
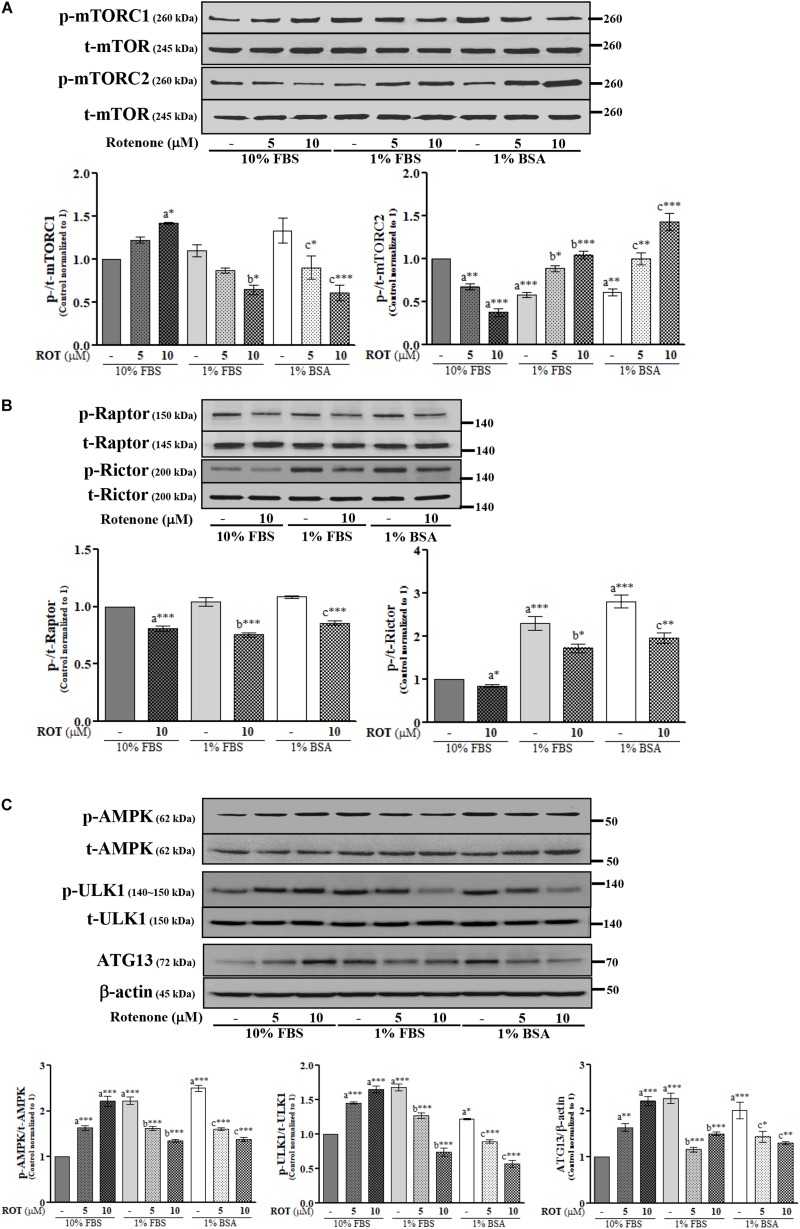
SH-SY5Y cells were seeded as 1 × 10^5^ cells/ml of DMEM containing 10% FBS or 1% FBS or 1% BSA and used for experiments after overnight incubation. Cells were incubated with the absence or presence of ROT (0, 5, and/or 10 μM) for 24 h and assessed for mTOR **(A,B)** and AMPK **(C)** signaling proteins and β-actin by Western blotting. Each picture is a representative of three independent experiments. Data are mean ± SEM of three independent experiments and analyzed by one-way of variance (ANOVA) followed by Tukey’s *post hoc* test. Statistical significance: _a_compared with 10% FBS control; _b_compared with 1% FBS control; _c_compared with 1% BSA control; ^∗^*p* < 0.05, ^∗∗^*p* < 0.01, and ^∗∗∗^*p* < 0.001.

The activities of mTORC1 and mTORC2 were assessed by measuring expression levels of phosphorylated (p)-mTORC1 (Ser2448) and p-mTORC2 (Ser2481), respectively. Control cells cultured in 1% FBS or 1% BSA exhibited slightly increased p-mTORC1 and significantly decreased p-mTORC2 (*p* < 0.001 and *p* < 0.01, respectively) (Figure 2A) compared to control cells cultured in 10% FBS. ROT (10 μM) dramatically increased p-mTORC1 (*p* < 0.05) and decreased p-mTORC2 (*p* < 0.001) expression in cells cultured with 10% FBS. Conversely, ROT decreased p-mTORC1 (*p* < 0.05 and *p* < 0.001, respectively) and increased p-mTORC2 (both *p* < 0.001) in cells cultured with 1% FBS or 1% BSA. These data suggest that FBS levels and ROT both alter mTOR signaling pathways in SH-SY5Y cells and that the effects of ROT differ depending on serum levels (nutrient availability). The overall effect of mTOR signaling depends on the specific complex activated, mTORC1 or mTORC2, which are distinguished by Raptor in mTORC1 and Rictor in mTORC2. Thus, we measured expression levels of p-Raptor (Ser792) and p-Rictor (Thr1135). Control cells cultured in 1% FBS or 1% BSA demonstrated no difference in p-Raptor expression but increased p-Rictor expression (*p* < 0.01 and *p* < 0.001, respectively) compared to control SH-SY5Y cells cultured in 10% FBS (Figure 2B), suggesting mTORC2 signaling predominance under low nutrient conditions. ROT treatment (10 μM for 24 h) decreased the expression levels of both p-Raptor (both *p* < 0.001) and p-Rictor (*p* < 0.05 in 10% and 1% FBS; *p* < 0.01 in BSA) in all three culture conditions, while total mTOR, Raptor and Rictor expression levels were unaffected (Figures 2A,B).

AMP-activated protein kinase is a major metabolic energy sensor that contributes to mTOR signaling through interactions with ULK1 and ATG13. Control cells cultured in 1% FBS or 1% BSA showed increased expression levels of p-AMPK (Thr172) (both *p* < 0.001), p-ULK1 (Ser757) (*p* < 0.001 and *p* < 0.05), and ATG13 (both *p* < 0.001) compared to control cells cultured in 10% FBS (Figure 2C). Treatment with ROT (10 μM for 24 h) significantly enhanced p-AMPK (Thr172), p-ULK1 (Ser757), and ATG13 expression by SH-SY5Y cells cultured in 10% FBS (all *p* < 0.001) but decreased expression levels of all three phosphorylated proteins in SH-SY5Y cells cultured with 1% FBS and 1% BSA, again indicating that serum influences mTOR signaling independently of ROT, and alters the mTOR signaling change in response to ROT.

### Rotenone Induces ER Stress and Disrupts MAM in SH-SY5Y Cells

**FIGURE 3:**
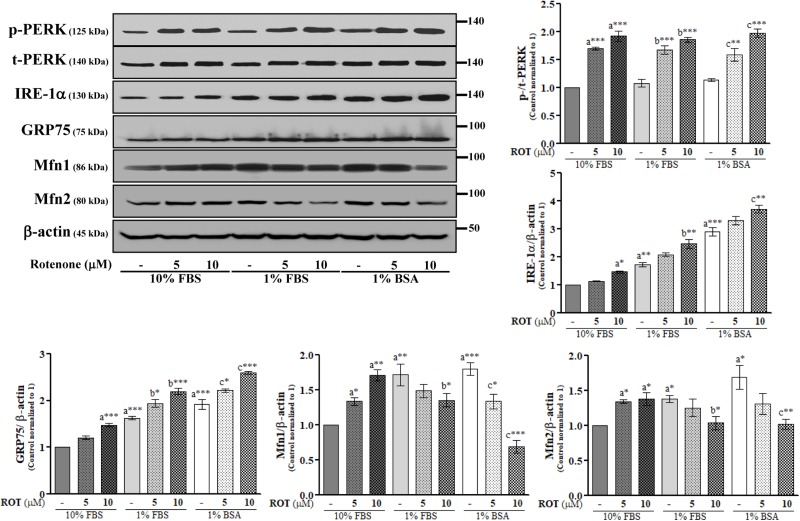
SH-SY5Y cells were seeded as 1 × 10^5^ cells/ml of DMEM containing 10% FBS or 1% FBS or 1% BSA and used for experiments after overnight incubation. Cells were incubated with the absence or presence of ROT (0, 5, and 10 μM) for 24 h and assessed for ER and MAM tethering proteins and β-actin by Western blotting. Each picture is a representative of three independent experiments. Data are mean ± SEM of three independent experiments and analyzed by one-way of variance (ANOVA) followed by Tukey’s *post hoc* test. Statistical significance: _a_compared with 10% FBS control; _b_compared with 1% FBS control; _c_compared with 1% BSA control; ^∗^*p* < 0.05, ^∗∗^*p* < 0.01, and ^∗∗∗^*p* < 0.001.

The endoplasmic reticulum is the central organelle responsible for protein folding, and there is compelling evidence that ER stress and protein misfolding are involved in ROT-induced PD-like toxicity. To examine ER stress in SH-SY5Y cells, we measured changes in the expression levels of protein kinase RNA (PKR)-like ER kinase (PERK) and inositol-requiring enzyme 1 α (IRE-1α). ROT (10 μM) increased expression of p-PERK at Thr981 (*p* < 0.001) and IRE-1α (*p* < 0.01) (Figure 3), suggesting that ER stress is involved in ROT-induced neuronal dysfunction. We further investigated the protein expression levels of MAM tethering proteins GRP75, Mfn1, and Mfn2. Control cells cultured with 1% FBS or 1% BSA showed significantly increased expression levels of GRP75 (both *p* < 0.001), Mfn1 (*p* < 0.01 in 1% FBS and *p* < 0.001 in 1% BSA), and Mfn2 (both *p* < 0.05) compared to control cells in 10% FBS (Figure 3). In addition, ROT (10 μM for 24 h) further increased the protein expression levels of GRP75 (*p* < 0.001) in all culture conditions. However, Mfn1 and Mfn2 were significantly increased by ROT (10 μM for 24 h) in cells cultured with 10% FBS (*p* < 0.01 and *p* < 0.05, respectively) but decreased in cells cultured in 1% FBS (both *p* < 0.05) or 1% BSA (*p* < 0.001 for Mfn1 and *p* < 0.01 for Mfn2).

### Intrastriatal Rotenone Injection Alters TH, PINK1, Parkin, and α-Syn Expression Levels in C57BL/6J Mice

**FIGURE 4:**
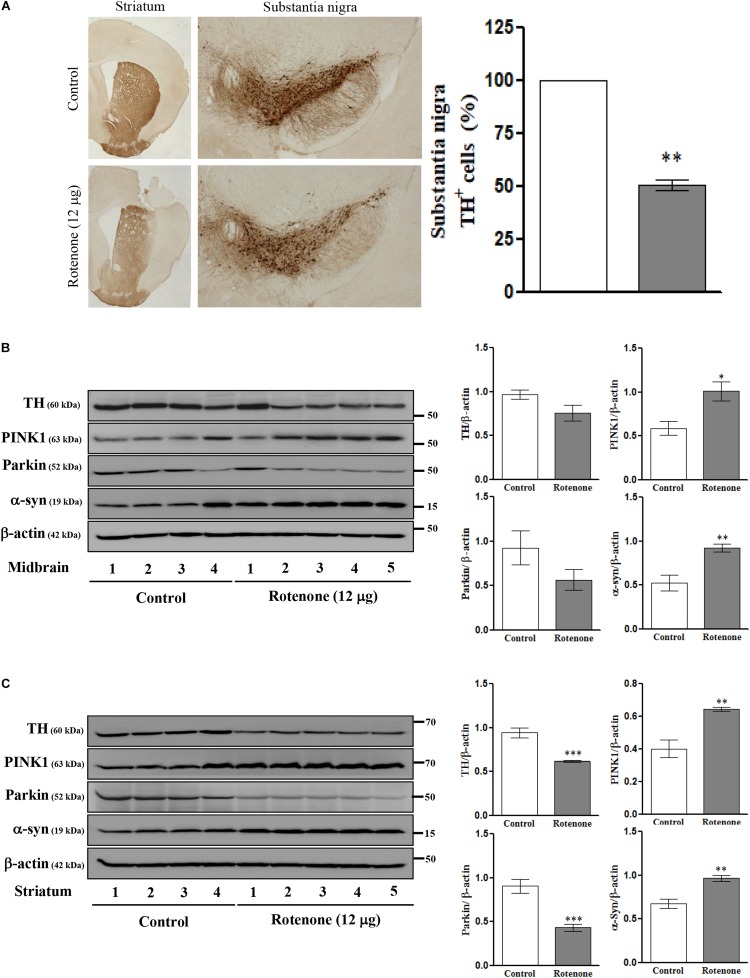
Photomicrograph of ROT lesioned C57BL/6J mice striatum and substantia nigra pars compacta (SNpc) immunostained for TH (25×). The quantitative image analysis of TH positive cells in SNpc expressed as percentage (%). Each picture is a representative of three independent experiments. Data are mean ± SEM (*n* = 3) and analyzed by paired two-tailed Student’s *t*-test. ^∗∗^*p* < 0.01 **(A)**. Effects of ROT on TH, PINK1, Parkin, α-syn, and β-actin in the midbrain **(B)** and striatum **(C)** of stereotaxic C57BL/6J mice were analyzed by Western blotting. Data are mean ± SEM (*n* = 4 for control; *n* = 5 for ROT) and analyzed by unpaired two-tailed Student’s *t*-test. ^∗^*p* < 0.05, ^∗∗^*p* < 0.01, and ^∗∗∗^*p* < 0.001.

To examine the effects of ROT on mTOR signaling, ER stress, mitochondrial function, MAM function, and cell viability *in vivo*, we conducted single unilateral intrastriatal ROT infusions (12 μg) in C57BL/6J mice. Instrastriatal injection induced, significant depletion of TH immunoreactivity in the striatum and SN (Figure 4A), and significantly reduced TH-positive cell numbers in the ipsilateral (injection-side) SNpc after 14 days (*p* < 0.01). The expression levels of TH and Parkin were decreased while PINK1 and α-syn expression levels were increased in midbrain (Figure 4B) and striatum (Figure 4C) of ROT-injected mice compared to vehicle (DMSO)-injected mice as measured by Western blotting, consistent with the effects of SH-SY5Y cells.

### Intrastriatal Rotenone Alters mTOR and AMPK Signaling in C57BL/6J Mice

**FIGURE 5:**
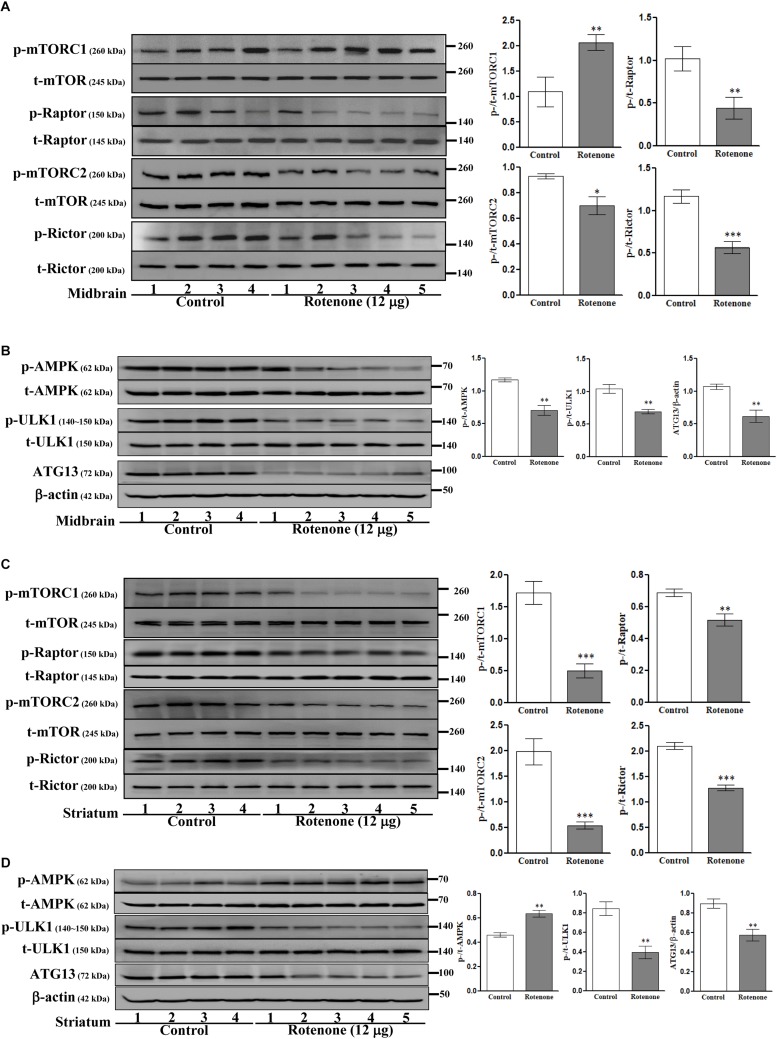
Effects of ROT on mTOR **(A,C)** and AMPK **(B,D)** pathways in the midbrain **(A,B)** and striatum **(C,D)** of stereotaxic C57BL/6J mice were analyzed by Western blotting. Data are mean ± SEM (*n* = 4 for control; *n* = 5 for ROT) and analyzed by unpaired two-tailed Student’s *t*-test. ^∗^*p* < 0.05, ^∗∗^*p* < 0.01, and ^∗∗∗^*p* < 0.001.

We then investigated the involvement of mTOR pathways in ROT toxicity. ROT injection increased p-mTORC1 (Ser2448) expression (*p* < 0.01) but reduced p-mTORC2 (Ser2481) expression (*p* < 0.05) in mouse midbrain (Figure 5A). Alternatively, ROT dramatically decreased both p-mTORC1 and p-mTORC2 in mouse striatum (*p* < 0.001; Figure 5C). In addition, p-Raptor and p-Rictor expression levels were reduced in the midbrain of ROT-injected mice (*p* < 0.01 and *p* < 0.001, respectively) (Figure 5A). Collectively, these findings suggest that ROT has region-specific effects on mTOR signaling pathways. Moreover, the protein expression levels of p-AMPK, p-ULK1, and ATG13 were decreased in ROT-injected mice (all *p* < 0.01), while t-AMPK and t-ULK1 expression levels remained unchanged (Figure 5B). In the striatum of ROT-injected mice, p-AMPK was increased while p-ULK1 and ATG13 expression levels were reduced (Figure 5D).

### Intrastriatal Rotenone Alters ER Stress and Disrupts MAM in C57BL/6J Mice

**FIGURE 6:**
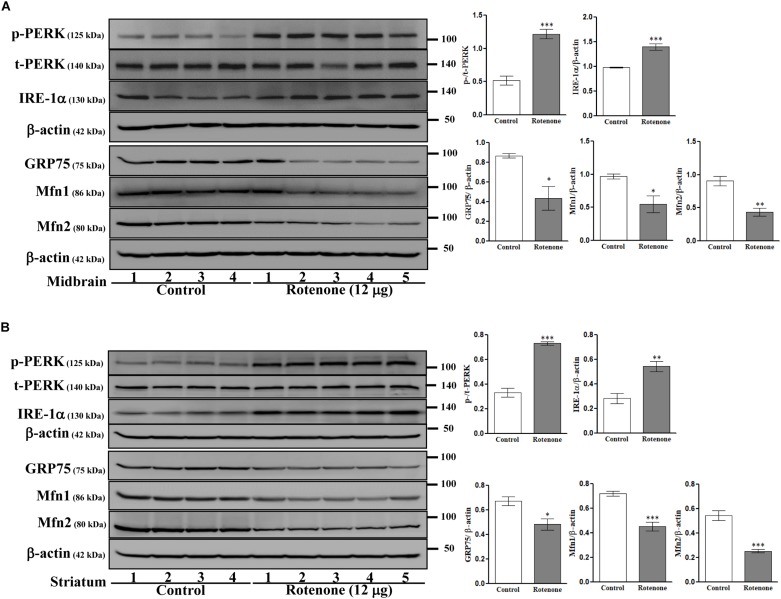
Effects of ROT on ER and MAM pathways in the midbrain **(A)** and striatum **(B)** of stereotaxic C57BL/6J mice were analyzed by Western blotting. Data are mean ± SEM (*n* = 4 for control; *n* = 5 for ROT) and analyzed by unpaired two-tailed Student’s *t*-test. ^∗^*p* < 0.05, ^∗∗^*p* < 0.01, and ^∗∗∗^*p* < 0.001.

To examine possible ROT-induced ER stress in the brain, we measured expression of the ER stress-associated proteins PERK and IRE-1α in midbrain (Figure 6A) and striatum (Figure 6B) lysates. Expression levels of p-PERK (Thr981) and IRE-1α were markedly increased by ROT in both midbrain and striatum (p-PERK: *p* < 0.001 in both regions; IRE-1α: *p* < 0.001 in midbrain, *p* < 0.01 in striatum) implicating ER stress in ROT induced neurodegeneration. Moreover, ER malfunction involved MAM dysfunction as the MAM tethering proteins GRP75, Mfn1, and Mfn2 were downregulated in midbrain (*p* < 0.05 for GRP75 and Mfn1; *p* < 0.01 for Mfn2) and striatum (*p* < 0.05 for GRP75; *p* < 0.001 for Mfn1 and Mfn2) of ROT-injected mice.

## Discussion

In recent years, many studies have been conducted to elucidate the molecular pathophysiology of PD progression. ROT, a fish poison that inhibits mitochondrial complex I, induces PD-like changes in cultured neurons, and rodent brain (Sherer et al., 2003). SH-SY5Y is a DA-producing human catecholaminergic neuroblastoma cell line widely used as an *in vitro* dopaminergic cell model (Martins et al., 2013). ROT treatment for 24 h dose-dependently reduced SH-SY5Y cell number. Moreover, serum starvation by culture in 1% FBS or 1% BSA medium inhibited cell growth and proliferation in the absence of ROT. Cells under serum starvation alone or with ROT detached from the culture surface and lost typical neuronal morphology within 24 h consistent with previous studies showing that serum starvation induces cell death (Chou and Yung, 1997) and that ROT-induced impairment of mitochondrial complex I activity leads to apoptosis, likely via excess ROS formation (Imamura et al., 2006).

Tyrosine hydroxylase, the rate-limiting enzyme in DA synthesis, is obviously critical for phenotypic expression (Nagatsu et al., 1964). ROT treatment for 24 h dramatically reduced TH expression in SH-SY5Y cells. Moreover, ROT infusion in mouse striatum reduced TH immunoreactivity and reduced TH-positive cell count in the SNpc after 14 days, consistent with the dopaminergic neuronal degeneration along the nigrostriatal pathway that parallels the symptoms of PD (Carriere et al., 2016). In addition, surviving cells accumulate damaged mitochondria, leading to metabolic deficits, oxidative stress, mitophagy, and greater susceptibility to other pathogenic processes such as protein aggregation (Shaltouki et al., 2015).

Parkin (PARK2), a cytosolic ubiquitin ligase, and PTEN-induced kinase 1 (PINK1; PARK6), a mitochondria-targeted kinase, act important mediators of mitochondrial quality control (Whitworth and Pallanck, 2017) by removing damaged or dysfunctional mitochondria and preserving a healthy mitochondrial population (Oh et al., 2017). Dysfunctional mitochondria can trigger cell degeneration via mitophagy in PD (Li et al., 2015). In the present study, ROT inhibited Parkin expression and enhanced PINK1 expression in SH-SY5Y cells as well as in mouse midbrain and striatum, suggesting that loss of coordinated Parkin/PINK1 function contributes to ROT-induced mitochondrial impairment, oxidative stress, and cell death. Loss of Parkin results in an initial accumulation of damaged mitochondria while PINK1 accumulation may cause proteosomal dysfunction (Wang et al., 2005; Shaltouki et al., 2015), which reduces Parkin solubility in toxin-induced PD models. Moreover, reduced Parkin in turn leads to the formation of protein aggregates resembling LBs in PD (Um et al., 2009, 2010).

Protein aggregation and filament formation are histopathological hallmarks of NDDs. For instance, PD is characterized by abnormal intracellular protein inclusions (LB and Lewy neurites or LN) mainly composed of aggregated α-syn fibrils (Faustini et al., 2017). The aggregation of α-syn causes proteasomal dysfunction, which may lead directly to neurodegeneration (Tanaka et al., 2001). We observed α-syn accumulation in SH-SY5Y cells and mouse midbrain and striatum following ROT treatment, consistent with previous studies demonstrating that ROT can trigger α-syn accumulation both *in vitro* and *in vivo* (Yuan et al., 2015). Aggregation of α-syn can be spread via a prion-like mechanism to neighboring neurons (Bae et al., 2012), resulting in the functional decline and death of dopaminergic neurons throughout the SNpc (Li et al., 2008; Lee et al., 2010). Moreover, α-syn can bind to TH, so accumulation of α-syn can reduce DAergic transmission by surviving cells (Perez and Hastings, 2004).

Under normal conditions, α-syn is cleared by proteolytic degradation in the extracellular space or lysosomal degradation in neighboring cells (Sung et al., 2005; Lee et al., 2008). However, impairment of these degradative mechanisms or an incapacity to clear proteins that have already aggregated is proposed the as primary defect leading to accumulation of insoluble α-syn (Cantuti-Castelvetri et al., 2005) within the LBs characteristic of PD (Myohanen et al., 2012), dementia with LB, and multiple system atrophy (Iwata et al., 2003). Previous *in vitro* studies have reported that the Triton-X100-soluble fraction represents bioavailable α-syn, whereas the Triton-X100-insoluble component may represent α-syn aggregated or sequestered in oligomeric forms (Klucken et al., 2003; Alves da Costa et al., 2006). In our present study, the oligomeric form of α-syn was detected only in the Triton-X100-insoluble fraction, while the monomeric form of α-syn was detected only in the Triton-X100-soluble fraction. ROT increased both Triton-X100-insoluble α-syn oligomers and Triton-X100-soluble monomer, consistent with previous studies reporting that ROT interacts with α-syn to drive accumulation of insoluble forms in SH-SY5Y cells (Sherer et al., 2002; Lee et al., 2004).

Growth factors and nutrients enhance protein synthesis and suppress protein degradation (Zhao et al., 2015). mTORs integrate signals from nutrients and growth factors with current energy status to regulate many neuronal processes, including autophagy, ribosome biogenesis, and growth (Sarbassov et al., 2005), as well as synaptic plasticity, learning and memory, and food uptake in adult brain (Zhou et al., 2015). Our present study suggests that both mTORC1 and mTORC2 regulate the responses to FBS and ROT in SH-SY5Y cells. ROT increased p-mTORC1 but decreased p-mTORC2 protein expression in the presence of 10% FBS, consistent with changes in the midbrain following ROT injection. Therefore, mTORC1 appears to be activated by ROT in the presence of sufficient nutrients and growth factors. The exact mechanism by which mTORC1 is activated and mTORC2 inhibited by ROT under this nutrient-rich condition requires additional studies. Control cells treated with 1% BSA also exhibited enhanced p-mTORC1 and reduced p-mTORC2 consistent with a previous study (Peruchetti et al., 2014). Conversely, ROT decreased p-mTORC1 and increased p-mTORC2 expression in the presence of 1% FBS or 1% BSA, changes shown to directly inhibit cell growth, and mitochondrial proteins during nutrient starvation. Our results are in line with another study reporting decreased mTORC1 in PC12 cells following ROT treatment for 24 h (Laplante and Sabatini, 2009). These results suggest that ROT treatment under nutrient shortage (serum starvation) inhibits mTORC1, resulting in lower mitochondrial membrane potential, oxygen consumption, and cellular ATP levels (Schieke et al., 2006).

Studies also suggest that mTORC1 acts as a negative regulator of mTORC2 (Dibble et al., 2009; Xie and Proud, 2014). Another study found that Rheb activated mTORC1 but inhibited mTORC2, while TSC1/2 inhibited Rheb/mTORC1 but activated mTORC2, most likely by overcoming the negative feedback loop (Yang et al., 2006). To further explore the role of mTOR complex signaling pathways in ROT toxicity, we examined expression of Raptor, a required cofactor for rapamycin-sensitive mTORC1 signaling, and Rictor, a regulatory subunit of mTORC2 (Li et al., 2007). ROT treatment decreased the phosphorylation levels of Raptor and Rictor both *in vitro* and *in vivo*, inconsistent with the aforementioned changes in mTORC1 and mTORC2 activity and suggesting that ROT can differentially stimulate or inhibit mTOR complex activities under different nutrient conditions; however, further investigations using specific substrates of these mTOR complexes necessary to examine the underlying mechanisms.

mTORC1 is the major transducer of nutrient signaling for cell growth (Efeyan et al., 2012). Similarly, AMPK senses energy deficiency in the form of an increased AMP/ATP ratio to regulate a myriad of cellular processes (Poels et al., 2009). The substrates ULK1 (ATG1) and ATG13 function downstream of mTORs and AMPK (Hosokawa et al., 2009; Egan et al., 2011). In this study and others (Wu et al., 2011), ROT-induced AMPK phosphorylation at Thr172 in SH-SY5Y cells cultured with 10% FBS medium. Increased p-AMPK was also reported in ROT treated HepG2 cells (Hou et al., 2018). Furthermore, activated ULK1 was shown to directly phosphorylate AMPK (Loffler et al., 2011). In contrast, Sciarretta et al. (2018) reported that ROT decreased mTORC1 and inhibited AMPK-ULK1 under serum starvation. Under starvation, AMPK translocates to the lysosome and both lysosomal AMPK and mTORC1 contribute to autophagy via ULK1/ATG13/FIP200 regulation (Ha et al., 2015). In this study, ROT inhibited mTORC1, AMPK/ULK/ATG13 in cells cultured with 1% FBS or 1% BSA (nutrient starvation). In ROT-injected mouse midbrain, the activation of mTORC1 by ROT inhibited the AMPK/ULK1/ATG13 pathway after 14 days. From the above results, we speculate that the nutrient-sensing molecules mTORC1 and AMPK/ULK1/ATG13 are differently regulated by ROT toxicity *in vitro* and *in vivo*.

The endoplasmic reticulum is involved in protein folding, maintenance of Ca^2+^ homeostasis and cholesterol synthesis, and ER dysfunction is implicated in the pathogenesis of α-syn mediated NDDs (Paillusson et al., 2017). The ER depends on ATP to correct misfolded protein errors, so ROT-induced ATP reduction can lead to ER stress, which in turn initiates the UPR through activation of PERK, and IRE-1α (Jiang et al., 2016). In the present study, ROT enhanced p-PERK Thr981 and IRE-1α expression levels, indicating ROT-induced ER stress, in both SH-SY5Y cells and mouse midbrain and striatum. This enhanced activation of PERK signaling results in a sustained reduction of global protein synthesis, leading to neuronal loss (Liu et al., 2015). In addition, PERK over-activation has been observed in post-mortem brain and spinal cord tissues of NDD patients (Smith and Mallucci, 2016). Taken together, the present study suggests that ROT-induced ER stress may trigger the death of SH-SY5Y cells and DAergic neurons in mice.

Mitochondria-associated ER membrane abnormalities have been described in cellular models of a number of NDDs, including PD (Cali et al., 2013). GRP75 is essential for maintaining physical contact between the ER and mitochondria, thereby facilitating Ca^2+^ exchange and transfer through ER-bound IP_3_R and mitochondrial VDAC1 (Honrath et al., 2017). Expression of GRP75 was increased in SH-SY5Y cells by ROT treatment but decreased in midbrain and striatum of C57BL/6J mice. As previously reported, GRP75 overexpression in human DAergic cells enhanced vulnerability to ROT-induced cytotoxicity (Jin et al., 2006), but *in vivo* GRP75 overexpression reduced infarct size and protected against mitochondrial damage in a rat middle cerebral artery occlusion model of stroke (Xu et al., 2009) and a rat model of intracerebral hemorrhage (ICH) (Lv et al., 2017). GRP75 expression was also decreased in the mitochondrial fraction isolated from the SNpc of PD patients compared to controls (Jin et al., 2006). The above results suggest that GRP75 may be either beneficial or harmful in different pathogenic contexts, although the exact mechanisms are still unknown (Shi et al., 2008). One possible explanation is provided by reports that GRP75 interacted with MAM-associated α-syn in a DAergic cell line with strong membrane attachment affinity due to a high lipid:protein ratio (Zabrocki et al., 2008; Fantini and Yahi, 2011).

The MAM tethering proteins mitofusions Mfn1 and Mfn2 are dynamin-related GTPases responsible for membrane fusion via a large cytosolic GTPase domain embedded in the outer mitochondrial membrane (Rojo et al., 2002; Suarez-Rivero et al., 2016). Mfn2 heterodimerizes with Mfn1 to link ER and mitochondria to regulate organelle tethering (de Brito and Scorrano, 2008; Chen et al., 2012). Our study suggests that α-syn can affect mitochondrial morphology and modulate mitochondrial dynamics through reduction of Mfn1- and Mfn2-dependent tethering in SH-SY5Y cells and mouse brain (Xie and Chung, 2012). Moreover, these findings identify MAM interface and inter-organelle contact disruption as novel mechanisms of ROT-induced α-syn toxicity.

Many studies rely on artificially overexpressed or recombinantly tagged proteins to investigate the underlying signaling mechanisms in PD. However, in the present study, we compared ROT-induced toxicity among cultures under different nutrient availability conditions and *in vivo* to further elucidate the potential mechanisms of α-syn neurotoxicity. As expected, ROT decreased TH and concomitantly increased α-syn accumulation, indicating degeneration of dopaminergic neurons both *in vitro* and *in vivo*. The nutrient-sensing mTOR and AMPK pathways appear to form a negative feedback loop that regulates the extent of ROT toxicity. More importantly, the present study implies that ER and MAM tethering proteins are also intimately involved in ROT-induced neurodegeneration. From these findings, we conclude that ROT-treated cell culture systems and mouse models are limited for recapitulating the clinical and pathological phenotypes of PD. We also conclude that serum concentration in culture medium can greatly influence the effects of ROT on cell growth, oxidative stress, and the various underlying signaling pathways. Further research *in vitro* and *in vivo* is necessary to establish stronger links between ROT-induced pathogenic mechanisms and human PD.

## Data Availability Statement

All datasets generated for this study are included in the manuscript/Supplementary Files.

## Ethics Statement

All animal experiments were approved by the Ethical Committee of Animal Research of the DGIST, Daegu, South Korea accordance with international guidelines.

## Author Contributions

MR and Y-IL conceived and designed the study, and wrote the manuscript. MR performed the major experiments. Y-JH helped to perform the animal experiments. MR analyzed the data. All authors read and approved the manuscript for publication.

## Conflict of Interest

The authors declare that the research was conducted in the absence of any commercial or financial relationships that could be construed as a potential conflict of interest.
