# Supplementary material for: The Impairments of α-Synuclein and Mechanistic Target of Rapamycin in Rotenone-Induced SH-SY5Y Cells and Mice Model of Parkinson’s Disease
Source: Front Neurosci. 2019 Sep 24;13:1028. doi: 10.3389/fnins.2019.01028 (PMC6769080; doi:10.3389/fnins.2019.01028)
Supplement: Supplementary file 1 [file Table_1.DOC]

**Title:**

**The Impairments of α-synuclein and Mechanistic Target of Rapamycin in Rotenone-induced SH-SY5Y Cell and Mice Models of Parkinson’s Disease**

Supplementary Table 1. List of antibodies used for Western blotting in this study.

| **Antibody Name** | **Host, MW Details** | **Company** | **Cat. No.** | **Dilution** |
| --- | --- | --- | --- | --- |
| *Primary Antibodies:* | | | | |
| Tyrosine Hydroxylase | Rabbit, 60 kDa | Novus | NB300_109 | 1:1,000 |
| PINK1 | Mouse, 63 kDa | Abcam | ab75487 | 1:2,000 |
| Parkin | Rabbit, 52 kDa | Abcam | ab15954 | 1:1,000 |
| α-synuclein | Mouse, 19 kDa | BD Transduction | BD_610786 | 1:1,000 |
| p-mTOR Ser2448 | Mouse, 220 kDa | Santa Cruz | sc-293133 | 1:500 |
| p-mTOR Ser2481 | Mouse, 220 kDa | Santa Cruz | sc-293132 | 1:500 |
| t-mTOR | Mouse, 245 kDa | Santa Cruz | sc-136269 | 1:2,000 |
| p-Raptor | Rabbit, 150 kDa | Cell Signaling | #2083 | 1:1,000 |
| t-Raptor | Mouse, 145 kDa | Santa Cruz | sc-81537 | 1:1,000 |
| p-Rictor | Rabbit, 200 kDa | Cell Signaling | #3806 | 1:1,000 |
| t-Rictor | Mouse, 200 kDa | Santa Cruz | sc-81538 | 1:1,000 |
| p-AMPK Thr172 | Rabbit, 62 kDa | Cell Signaling | #2535 | 1:1,000 |
| t-AMPK | Rabbit, 62 kDa | Cell Signaling | #2532 | 1:1,000 |
| p-ULK1 Ser757 | Rabbit, 140~150 kDa | Cell Signaling | #6888 | 1:2,000 |
| t-ULK1 | Rabbit, 150 kDa | Cell Signaling | #8054 | 1:2,000 |
| Apg13 | Rabbit, 72 kDa | Cell Signaling | #6940 | 1:2,000 |
| p-PERK Thr981 | Rabbit, 125 kDa | Santa Cruz | sc-32577 | 1:500 |
| t-PERK | Rabbit, 125 kDa | Cell Signaling | #3192 | 1:2,000 |
| IRE-1α | Rabbit, 130 kDa | Cell Signaling | #3294 | 1:2,000 |
| GRP75 | Rabbit, 75 kDa | Cell Signaling | #3593 | 1:2,000 |
| Mitofuscin 1 | Mouse, 86 kDa | Santa Cruz | sc-166644 | 1:500 |
| Mitofuscin 2 | Rabbit, 80 kDa | Cell Signaling | #9482 | 1:5,000 |
| β-actin-HRP | Mouse, mAb, 45 kDa | Cell Signaling | #5125 | 1:20,000 |
| β-actin | Mouse, mAb, 42 kDa | Sigma | A1978 | 1:20,000 |
| *Secondary Antibodies:* | | | |  |
| Bovine anti-rabbit IgG-HRP | Rabbit | Santa Cruz | sc-2370  sc-2357 | 1:2,000  ~1:10,000 |
| Bovine anti-mouse IgG-HRP | Mouse | Santa Cruz | sc-2371  sc-516102 | 1:2,000  ~1:10,000 |
