# Supplementary material for: The Impairments of α-Synuclein and Mechanistic Target of Rapamycin in Rotenone-Induced SH-SY5Y Cells and Mice Model of Parkinson’s Disease
Source: Front Neurosci. 2019 Sep 24;13:1028. doi: 10.3389/fnins.2019.01028 (PMC6769080; doi:10.3389/fnins.2019.01028)
Supplement: Supplementary file 2 [file Presentation_1.PPT]

## Slide 1
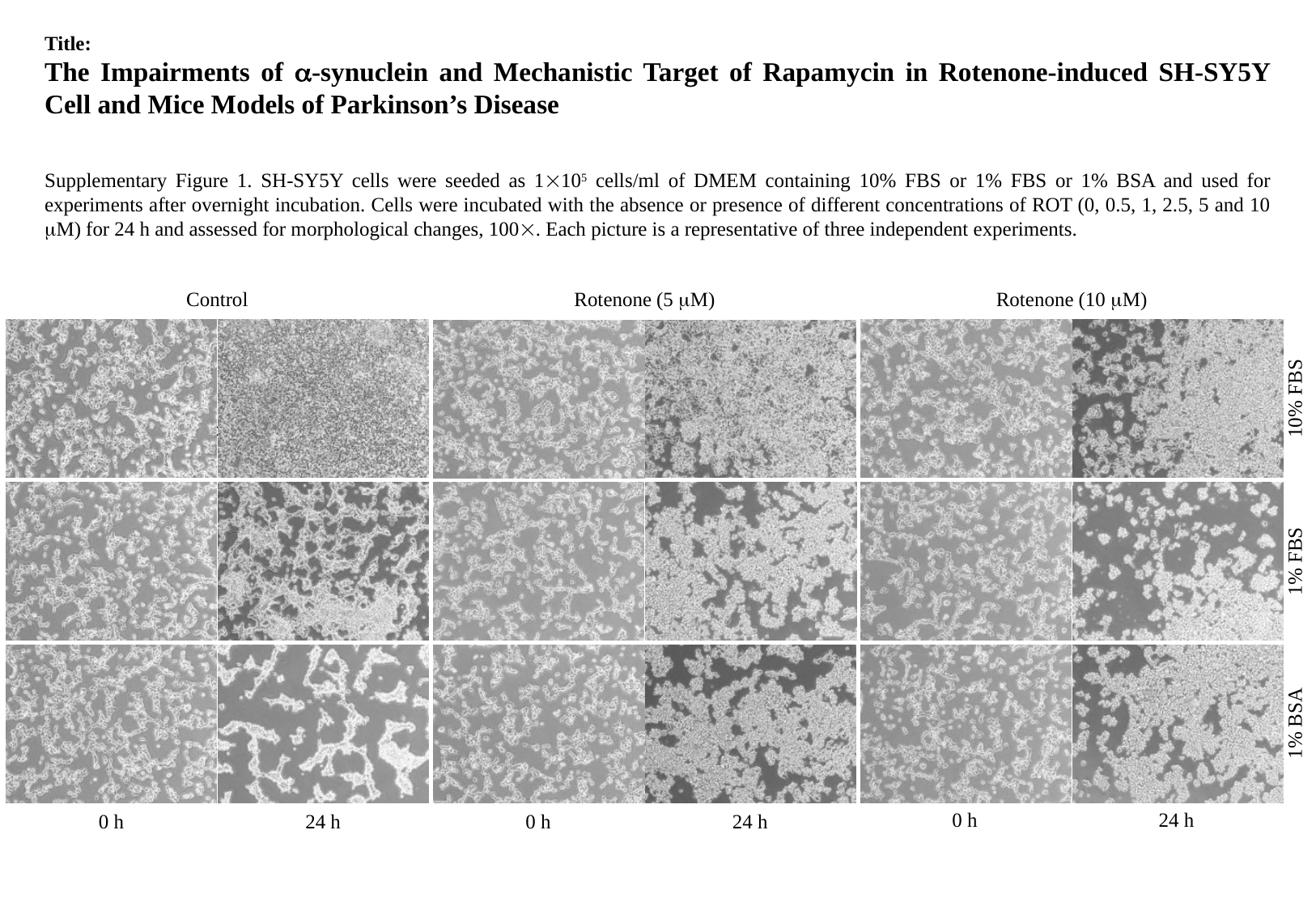

Title:
The Impairments of α-synuclein and Mechanistic Target of Rapamycin in Rotenone-induced SH-SY5Y Cell and Mice Models of Parkinson’s Disease
Supplementary Figure 1. SH-SY5Y cells were seeded as 1×10^5^ cells/ml of DMEM containing 10% FBS or 1% FBS or 1% BSA and used for experiments after overnight incubation. Cells were incubated with the absence or presence of different concentrations of ROT (0, 0.5, 1, 2.5, 5 and 10 μM) for 24 h and assessed for morphological changes, 100×. Each picture is a representative of three independent experiments.
Control
Rotenone (5 μM)
Rotenone (10 μM)
10% FBS
SUPPLEMENTAL FIGURE 1.
1% FBS
1% BSA
0 h
24 h
0 h
24 h
0 h
24 h
